# Railway Accidents

**Published:** 1907-02

**Authors:** 


### RAILWAY ACCIDENTS.

One of the unpleasant experiences of the rush of the Twentieth Century is that accidents are greatly increased in proportion to what they were say twenty-five years ago, and yet these accidents cause no apparent excitement in the community, no general attempt to make things better. Railway trains run through drawbridges that have not been properly closed, or are thrown off going around a cure at an exceedingly rapid rate, a great many people are killed but hardly anyone thinks any more about it than what is embraced in reading the morning paper. Last summer a writer in the Outlook took up this matter and attributes many of the accidents occurring in railways to the fact that especially on single-track roads, the freight train crews are on duty as great a number of consecutive hours as fifty-nine. It is easy to understand what kind of service one could rely on with a crew of men who have been working in this way. According to the writer who is quoted by the Boston Medical and Surgical Journal, it seems that this "sort of thing is not at all uncommon during times of activity in the transportation of freight, particularly where rival lines are concerned." It was a personal observer who is writing this, and we quote a very small part of his important words:

"Nobody will question the necessity of a good night's rest to the performance of keen, accurate, and efficient work, and yet how many people are here to-day who realize that the freight train crews of our railroads, especially in winter and on single-track lines, are often on duty for twenty-four to thirty-six hours without sleep. The artisan, the laborer, the miner, the mill-hand and the clerk work but ten hours at the most during the twenty-four, and yet the men who share with the eight-hour trick despatchers the responsibility for the safety of the traveling public, rarely doff their overalls short of the sixteen-hour mark. They are paid overtime—of course they are—and at an increased rate per hour or per mile; but ask a dozen engineers and freight conductors whether the hardship of overtime is counterbalanced by the extra wages, and, unless some member of the group is trying to pay off a mortgage on a neat little cottage and lot, every man's answer will be an emphatic negative. Work that is paid for in blood should be prohibited, and the toilers supplanted by fresh, wide-awake comrades."—The Post-Graduate, Dec., '06.

